# Determinants of Physical Activity Performed by Young Adults

**DOI:** 10.3390/ijerph16214061

**Published:** 2019-10-23

**Authors:** Jacinto García-Fernández, José Rafael González-López, Ángel Vilches-Arenas, María de las Mercedes Lomas-Campos

**Affiliations:** 1 Nursing Department, Faculty of Nursing, Physiotherapy and Podiatry, Universidad de Sevilla, 41009 Seville, Spain; jacintogf@us.es (J.G.-F.); mlomas@us.es (M.d.l.M.L.-C.); 2 Preventive Medicine and Public Health Department, Faculty of Medicine and Faculty of Nursing, Physiotherapy and Podiatry, Universidad de Sevilla, 41009 Seville, Spain; ava@us.es

**Keywords:** health status, health promotion, healthy lifestyle, leisure time, work, Spain

## Abstract

Despite the World Health Organization considering it important to promote physical activity as part of a healthy lifestyle, the official data show an increase in the percentage of physical inactivity, which has brought about the development of strategies at different levels (national and international) to reverse this trend. For the development of these strategies, it is relevant to know what the determinants of physical activity (at leisure and at work) are. Therefore, this is going to be analysed in the autochthonous young adults from Seville. A cross-sectional survey of their health behaviours was carried out. The sample was selected through a proportionally stratified random sampling procedure. From the results, we highlight that the general perceived health status is good and that most physical activity is performed during leisure time. However, a majority of the population analysed reported overweight or obesity. Participants with a low perceived health status, those who have low social support from their family and friends, and those who do not smoke are the ones who have more probability of engaging in physical activity during their leisure time. However, gender, education level, and alcohol consumption are revealed as determinants of the intensity of physical activity at work. In this regard, men and/or participants with a low level of studies are those who carry out more physically demanding activities at work.

## 1. Introduction

Physical inactivity has been noted as the second leading modifiable risk factor for chronic disease (after smoking), and contributes significantly to total mortality in Western countries [1,2]. In addition, it is an important component affecting obesity-related chronic disease and the ability to maintain weight [3,4]. For all these reasons, the World Health Organization (WHO) considers it important to promote physical activity (PA) as part of a healthy lifestyle [5]. PA is defined by Caspersen et al. [6] as “any bodily movement produced by skeletal muscles that results in energy expenditure.” That includes not only practising sports but also occupational, conditioning, household, or other activities [6]. 

Despite the importance of exercise or sport in health, the percentage of people from the EU who engage in some kind of exercise or sport has decreased in recent years, with the last reported level of inactivity in the EU being 46% [7]. The data from Spain are quite close to the EU values, so the percentage of people who practise some kind of sport or exercise has been decreasing since 2009. 

For a decade, national and European strategies [8,9] have been devised to try to reverse this trend, so it is necessary to know what the determinants of performing PA are. Furthermore, PA interventions targeted at sedentary healthy adults are cost-effective [10]. There are, however, few studies about PA in young adults in contrast to other life cycle stages such as childhood, adolescence, and old age [11,12,13].

On this subject, Baunan et al. [14] proposed an ecological multilevel model of PA—individual, social, environmental and policy levels are considered—since the combination of different levels of factors is expected to influence the level of PA. In this model, they pointed out that the importance of each determinant of PA depends on the life cycle stage. In this case, determinants that theoretically have more influence in young adults are those related to the environment and policy, although individual and social factors may also have a significant effect on the level of PA of this group. 

Previous research [15,16,17,18,19,20] has analysed demographic (gender, age, education), biological (health status, BMI), as well as social (support from family and friends) variables as determinants of PA in adults; however, the results are not homogeneous since the country in which the study is carried out plays an important role [7]. 

In this respect, according to the statistics [7] the percentage of people who perform PA varies according to age, gender, and education level. In this sense, the percentage of inactivity increases with age [21]. Therefore, while the level of inactivity in adolescents is 19%, the number of people aged 55 or over who do not carry out any kind of exercise is 58% [7]. Regarding gender, differences between men and women among adults are reported [22,23,24,25,26,27]. According to the WHO [28], women perform less PA than men. Nevertheless, women have a stronger adherence to PA recommendations than men [29,30]. As to the education level [7], the results revealed that if people left the educational system by the age of 15 or earlier, their inactivity level is higher (68%) than if they finished their education at the age of 20 or over (27%). According to the Programme for the International Assessment of Adult Competencies (PIACC) report [31], the percentage of people from Spain who did not finish secondary school in 2013 was around 47%, which is almost double the average rate in Europe (26%).

In addition, PA also depends on the perceived health status and the body mass index (BMI) category [18,32]. Considering that improving health status and weight control are the most common reasons for engaging in PA [7], it is expected that people with a lower level of self-perceived health and/or with a higher BMI are those who are more likely to perform PA.

Based on the data from the Ministry of Health, Social Services and Equality in Spain [33], 71% of the population perceived their health status as being very good or good. According to Eurostat [34], in 2017 the population the European population aged 18 or over that was overweight (BMI equal to or greater than 25) was 52.0% and for obesity it was 15.2% (BMI equal to or greater than 30); the evidence is similar in Spain. BMI is also associated with national cultural factors [35], education level, and socioeconomic status [36].

Alcohol and tobacco consumption are not considered to be health-promoting behaviours [37,38]. Some research [39,40] has described people who are physically active as also being likely to be moderate drinkers. Dodge et al. [41] relate high-intensity aerobic PA with a protective effect of alcohol compared with other kinds of PA. With respect to tobacco, some authors have found a negative association between PA and smoking [42].

Focusing on interpersonal variables, family [43] and friend [44] support has also been shown to have a positive association with PA. This is defined as “aid and assistance exchanged through social relationships and interpersonal transactions” [45]. As was argued by Scarapicchia et al. [46], although there is previous empirical evidence, it is inconsistent and so more research should be carried out in this area. 

Considering that PA is a complex behaviour [47], and the previous inconclusive research and scarcity of studies focused on young adults, it is important to go deeper into the different determinants of PA. Therefore, the aim of this research is to study the determinants of PA carried out by autochthonous young adults from Seville. 

## 2. Materials and Methods

### 2.1. Design and Sample

A cross-sectional survey of health behaviours of young adults from Seville was carried out. Native adults aged 25–44 years old and living in Seville were invited to take part, because the objective was to analyse the behaviour of autochthonous inhabitants. Seville has 702,355 inhabitants and is the capital of the Regional Community of Andalusia, Spain. Data collection was carried out in Seville’s 11 administrative districts, from January to May 2012. To obtain a representative sample, a proportionally stratified random sample was used, which took people’s distribution by district, gender, and age into account. The minimum sample size was established at 383 people, following the criteria of González-López et al. [32]: (a) the number of adults included in the census in Seville, (b) a 95% reliability level, and (c) a 0.86% probability of finding some of the health behaviours studied. 

Inclusion criteria were men or women (a) born and residing in any of the official neighbourhoods or census sections from the 11 administrative districts in Seville; (b) 25–44 years old; (c) able to communicate and understand the requirements of the study; and (d) who signed the informed consent document. The main exclusion criterion was experiencing a mental illness that prevented them from understanding the purpose of the study. The total number of people interviewed was 409, due to the mortality of the sample (around 7%).

Most of the respondents were contacted directly by the interviewer on the street or while waiting outside primary care centres. Potential participants were approached and informed of the aim of the study. The facts that participation was anonymous, that data collection and handling were confidential, and that the survey results might serve to improve the provision of educational programmes and health services were highlighted.

### 2.2. Instrumentation

Sections of the previously validated Behavioural Risk Factor Surveillance System questionnaire [48,49] were used. The questionnaire was read aloud, and the answers were tabulated by the interviewer. 

PA variables included in the research are described in Table 1. While doing leisure time physical activity (LTPA) on a regular basis and the level of PA at work were used such as dependent variables of the regression analysis, the frequency and types of LTPA as well as the types of PA at work were used for descriptive purposes. 

In addition, socio-demographic characteristics (gender, age (from 25 to 44 years old), education level (without studies; primary, secondary, or university), employment status (employed, unemployed, or other situations), and district), self-perceived level of health (ranked from 1 to 5, from poor to excellent), BMI as calculated considering the height and weight self-reported by participants and classified following the protocol used for the DORICA study (Spanish Society for the Study of Obesity) [50] (normal weight, overweight and obesity), social support from friends and family (ranked 1 to 5, from always to never), current alcohol consumption (Yes/No, depending on whether the participant had drunk alcohol in the last 30 days), current tobacco consumption (Yes/No) were considered as independent variables in our analysis. 

### 2.3. Ethical Considerations

All subjects gave their informed consent for inclusion before they participated in the study. Data collection was carried out exclusively by an interviewer. Consent forms and questionnaires were stored in a locked container. The data were transferred to a password-protected computer database. The study was conducted in accordance with the Declaration of Helsinki of 1975 (updated in 2013), and the protocol was approved by the Ethics Committee of the University of Seville (12/05/09). 

### 2.4. Statistical Analysis

The data were analysed with the IBM SPSS Statistics package, version 25, for Windows (IBM, Armonk, NY, USA). The findings were reported using descriptive statistics. In addition, Logit and ordinal regression (depending on the characteristics of the dependent variable in each case) were tested. The models are specified as follows:

Model 1 and 2: LTPA = ß1+ ß2 Gender + ß3 Age + ß4 Employment + ß5 Health Status (BMI categories or self-perceived level of health) + ß6 Social Support + ß7 Alcohol + ß8 Tobacco + ε 

Model 3 and 4: Intensity of PA at work = ß1+ ß2 Gender + ß3 Age + ß4 Education level + ß5 Health Status (BMI categories or self-perceived level of health) + ß6 Social Support + ß7 Alcohol + ß8 Tobacco + ε 

Considering the majority of the variables are qualitative, some of them have been recoded to facilitate their inclusion in the models, such as the dummy variables employment (1 if the participant has a paid job, 0 otherwise), education level (1 if the participant has at least higher studies, 0 otherwise) and self-perceived health level (1 if excellent, very good or good, 0 if fair or poor). 

## 3. Results

### 3.1. Sample Demographic Characteristics and Health Status

The sample was composed of 205 (50.1%) men and 204 (49.9%) women. The average age was 34.7 years (SD = 7.0; [95% CI: 34.0–35.3]). Almost half of the participants had completed a university (43.5%) or secondary school level education (42.3%). Two-thirds of the sample were employed (62.6%); 84.8% of autochthonous young adults from Seville perceived their health status as good, very good, or excellent (28.4%, 37.6%, and 18.8%, respectively), compared to 15.2% who perceived it as bad (1.0%) or fair (14.2%). The BMI of the studied population ranged between 19.1 and 47.1 kg/m^2^, with an average of 28.6 kg/m^2^ (SD = 3.9); almost 50% were classified as overweight and 33.7% were classified as obese. Only 16.4% of the sample reported a normal weight. 

In relation to social support, 59.9% always or almost always received support from their partner. Almost two-thirds of the sample always or almost always had support from their family or friends; 37.9% of the sample never received support from other people. With respect to current alcohol consumption, 87% of the participants had consumed alcohol in the last 30 days. Currently 194 people (47.4%) smoked cigarettes and of them 134 (67.0%) smoked daily.

### 3.2. Physical Activity

**Figure 1:**
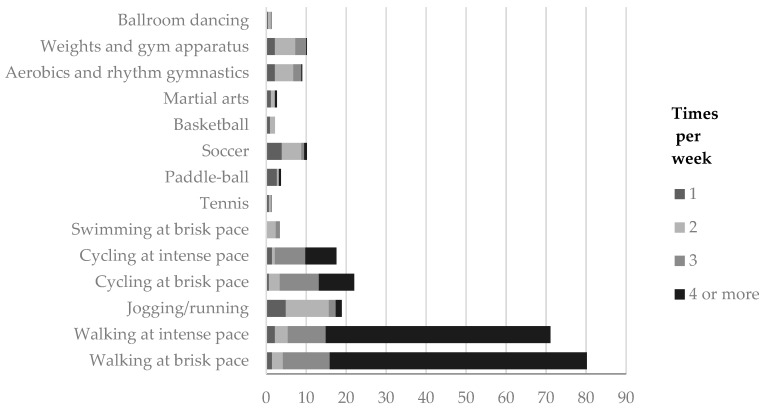
Types of LTPA of at least 30 min.

In this study, 78.5% of the people interviewed had engaged in some type of LTPA during the week before the survey was conducted; 38.6% of the sample reported that they carried out a single type of LTPA. The percentage decreased when the number of different PA activities increased; consequently, only 17.8% of autochthonous young adults from Seville performed at least three types of PA. A description of the types of PA can be seen in Figure 1. From these results, we highlight that four or more times a week 64.3% of the sample walked at a brisk pace and 56.2% walked at an intense pace. Furthermore, 10.8% of the participants went running twice per week; 9.8% practised cycling at a brisk pace three times a week. In addition, 4.9% of the sample played soccer twice a week, 4.6% practised aerobics and rhythmic gymnastics, and 5.1% used weights and gym apparatus with the same weekly frequency.

Moreover, 80.1% of the respondents reported low PA at work, compared to 19.9% who said that their activity was intense (18.7%) or moderate (1.2%). The most frequently reported physical activities at work were standing or sitting (71%), followed by walking (17.9%), and transporting light loads or frequently going up stairs or inclines (3.9%). Only 1.8% referred to performing heavy work that demands a lot of effort. 

Table 2 shows the results of the regressions so we can see what the determinants of LTPA are. Social support from family and friends, and alcohol and tobacco consumption, had a statistically significant impact on the probability of carrying out LTPA. Considering that the social support variable is inverted, people who had less social support were more likely to engage in LTPA. While alcohol consumption had a positive impact on the likelihood of performing LTPA, tobacco consumption had a negative effect. Although the results on alcohol consumption are surprising, we must point out that this variable only considers whether the participant had consumed some alcohol in the last 30 days; it does not reflect whether their consumption was excessive or not.

In addition, the health status variables’ results differed. Whereas the different BMI categories did not indicate any significant impact on the LTPA, the self-perceived level of health had a statistically significant, negative effect. Considering how this variable is defined, these results imply that people with a lower self-perceived level of health are more likely to carry out LTPA.

The determinants of the level of PA at work are presented in Table 3. Education level and gender are revealed as the variables that most influence the level of PA at work. People with a higher level of education perform low-intensity PA at work, while male gender implies that the kind of physical activities required at work are more intense. In addition, alcohol consumption presents a statistically significant, negative impact on the intensity of PA at work. The participants who had not drunk alcohol in the last 30 days perform more intense physical activities at work, while those who consumed alcohol in this period carry out less intense physical activities. Finally, the health status did not influence the intensity of PA carried out at work.

## 4. Discussion

According to the results, the average profile of young autochthonous adults in the city of Seville and, therefore, in the sample, was: male or female, with an average age of 35 years old, having university studies or secondary education, who performs a paid job and perceives their health status as good, very good, or excellent (especially men) and is overweight. Regarding PA, their work did not require very intense PA, while most of them declared that they performed LTPA.

With regard to LTPA, the percentage of people who did no PA at all, or did not do PA regularly (just over 21.5%), was close to that established by the Andalusian health survey (25.3%) in the same age range [51], and lower than in the Spanish survey (around 30–35%) of the adult population of 18 to 49 years old [26], or 58% of people aged 55 or over, published more recently by the European Commission [7]. There are no data regarding other studies in our city, so we cannot interpret the figures according to a trend, in a similar way to that established by Redondo et al. [52] in Gerona, by the SIVFRENT-A study in Madrid [25,53], or by the Spanish national survey [54]. In our study, the likelihood of carrying out LTPA was not influenced by age. However, previous research [7,21,51] has supported the notion that inactivity increases with age. In this sense, it is necessary to point out that the sample does not include older people, who are usually reported as the group with the highest level of inactivity.

Although some previous evidence supports gender having an influence on the probability of engaging in LTPA [15,16], our findings suggest that this is not a statistically significant relationship. However, as Artazcoz et al. [55] argue, “the gender differences in activity in leisure time can be explained by cultural patterns that encourage more sports among men, but also by the time constraints related to greater occupation of women (work and housework).” In our study the fact of being employed or not had no effect on the probability of performing LTPA. In this sense, Päivärinne et al. [56] described an inverse linear relationship between LTPA and the number of years working.

When we related LTPA to the level of self-perceived health, our data showed a statistically significant, negative effect. This can be explained by people who generally have a lower perception of their health being those who report a higher probability of engaging in PA, since they consider PA as the way to improve their health. This is also supported by recent evidence from the USA [57]. On the contrary, the self-perceived health level had a positive, direct effect on LTPA in Finnish people aged 46 years old [58]. The difference between these results and those obtained by our analysis could be due to the sample composition and the cultural impact.

Focusing on the social support variable, our findings suggest an inverse relationship with the practice of LTPA. This may be due to the fact that people who have less support perform PA to curry social relationships [7]. As examples, various studies in people over 44 years old [43,44,59] state that there is a direct relationship between the support of family and friends and the performance of PA.

Alcohol consumption has a positive impact on the likelihood of carrying out LTPA. In this sense, we need to clarify that this measure does not have any negative connotation since it only records whether the participant has consumed alcohol in the last 30 days. Alcohol consumption can simply be considered as another way of socialising, which could be due to celebrating with peers reaching some kind of objectives [39,40,47]. However, our findings reveal an inverse impact of tobacco consumption on LTPA. Our data from young adults are in line with those reported by Kwan et al. [60]. Other research [61,62] also describes the negative association between PA and smoking, since smokers have a limited lung capacity, which means that PA is more difficult for them.

With regard to PA at work, gender and educational levels show a significant influence on the level of PA. As to gender, we argue that the most demanding physically intense jobs are usually carried out by men, which implies gender inequality [63]. In addition, Pulakka et al. [64] also report that PA differs by gender during working time.

Our data (80.1%) on the low intensity of PA at work showed a somewhat lower percentage than those offered by health surveys in Andalusia (82.5%) in the same age stratum [51], and in Spain (82.6%) in the population between 25 and 64 years old [26]. These differences are possibly related to the job of the people surveyed. Yet they are not significant enough for us to be able to establish any hypothesis on this subject.

In this regard, our results reveal that a higher educational level is related to a lower level of PA at work. If the level of studies is higher, this person will probably be in charge of other, less physically demanding duties.

Finally, a negative impact of alcohol consumption on PA at work is evident. The main reason may be responsibility about its consumption and subsequent working hours, as well as the intensity of the PA. We have not found a relationship between the level of PA at work and tobacco consumption or social support.

### Limitations and Future Research Directions

The data were based on self-reports and were not verified using other methods. In this respect, the level of PA at work was a self-reported measure, although participant perception may depend on an individual’s previous exercise experience and relative level of fitness. Future research on this topic should control the self-reported measures with objective data such as Metabolic Equivalent of Tasks (METs).

Although the cross-sectional design allowed us to establish some factors that have an influence on the PA, they are mainly based on the individual at the social level of the model proposed by Bauman et al. [14], so it is necessary to go further in the analysis of the determinants of PA by considering variables from the environment and policy levels.

Since the results obtained are very sensitive to a country culture’s, it would be relevant to carry out this kind of study in other contexts in order to be able to develop specific public health policies that encourage PA.

## 5. Conclusions

At the beginning of our research, we stated that our objective was to go further into the determinants of PA (at leisure and at work) carried out by autochthonous young adults from Seville.

A majority of participants performed LTPA, with walking, cycling, and running being the most popular activities. Based on the findings, the participants engaged in LTPA mainly due to a poor self-perceived state of health or for socialisation purposes. In addition, smokers less often perform LTPA.

In general, the job activities of participants were not very physically demanding, although more intense activities were carried out by men and/or people with a low education level. Furthermore, people who consumed alcohol carried out less intense PA at work.

As the level of physical inactivity reported in young adults was lower than in other contexts, different city institutions should encourage PA due to its importance for health; therefore, they should develop some strategies to achieve lower levels of physical inactivity. Therefore, specific strategies should be implemented for each age group (adolescents, young adults, and older people), since their characteristics are different.

For the development of these strategies in young adults, the insights from this research could be useful since the main determinants of PA are reported.

## Figures and Tables

**Table 1:** Descriptions of PA variables.

Does Leisure Time Physical Activity (LTPA) on a Regular Basis	Dummy Variable (Yes/No)
Types of LTPA	Yes, if it was carried out at least three times a week for at least 30 min: walking at a brisk pace, walking at an intense pace, jogging/running, cycling at a brisk pace, cycling at an intense pace, swimming at brisk pace, tennis, paddle-ball, soccer, basketball, martial arts, aerobics and rhythmic gymnastics, weights and gym apparatus, ballroom dancing, other PA.
Frequency of LTPA	None, one, two, three, or more than three times per week during the week before the interview.
Types of PA at work	Standing, sitting, walking, carrying light loads, climbing stairs or going up inclines, hard work.
Level of PA at work	Low, moderate, intense.

**Table 2:** Results of logit regression of LTPA.

Variable	Model 1	Model 2
Constant	1.579	4.599 *
Gender	−0.694	−0.504
Age	−0.035	−0.055
Employment status	0.638	0.632
Self-perceived level of heath		−2.273 *
BMI		
Overweight	−0.081	
Obesity	−0.0195	
Social support from friends and family	0.857 ***	0.619 *
Alcohol	1.734 ***	2.007 ***
Tobacco	−1.736 ***	−1.981 ***
Likelihood ratio statistics χ²	40.13 ***	46.06 ***

Significance level: *** *p*-value < 0.005, ** *p*-value < 0.01, * *p*-value < 0.05.

**Table 3:** Results of logit regression of level of PA at work.

Variable	Model 3	Model 4
Gender	1.559 ***	1.405 ***
Age	−0.011	0.009
Education level	−1.100 ***	−1.147 ***
Self-perceived level of health		0.766
BMI		
Overweight	−0.404	
Obesity	0.458	
Social support from friends and family	0.237	−0.217
Alcohol	−1.718 **	−1.488 *
Tobacco	−0.096	0.175
Likelihood ratio statistics χ²	39.36 ***	35.63 ***

Significance level: *** *p*-value < 0.005, ** *p*-value < 0.01, * *p*-value < 0.05.
